# Decreasing incidence and severity of Behçet’s disease: a changing trend in epidemiological spectrum possibly associated with oral health

**DOI:** 10.3906/sag-2003-147

**Published:** 2020-11-03

**Authors:** Gonca MUMCU, Fatma ALİBAZ-ÖNER, Tülin ERGUN, Haner DİRESKENELİ

**Affiliations:** 1 Faculty of Health Sciences Marmara University, İstanbul Turkey; 2 Department of Internal Medicine, Division of Rheumatology, School of Medicine, Marmara University, İstanbul Turkey; 3 Department of Dermatology, School of Medicine, Marmara University, İstanbul Turkey

**Keywords:** Behçet’s disease, epidemiology, oral health

## Abstract

Behçet’s disease (BD) is a systemic and inflammatory disorder that is mainly present along the ancient Silk Road, from the Mediterranean Sea to East Asia. A wide range of prevalence figures (0.1–420/100,000) have been reported for BD, also among Turkish populations of similar genetic background living in different countries. Recently, a decline in the incidence of BD and a change of the disease spectrum to less-severe manifestations have been reported from Japan and Korea, two genetically homogenous, affluent populations with limited immigration. It was hypothesized that a decline in infectious diseases, especially dental/periodontal infections, associated with the improvement in oral health, could be a part of these changes in the disease expression. Further epidemiological studies in other populations might demonstrate whether there is a worldwide similar trend and may provide a better understanding of the triggering factors for the onset and course of BD.

## 1. Introduction

Behçet’s disease (BD) is a systemic, inflammatory disorder of unknown etiology. It is characterized by oral and genital ulcers, cutaneous, ocular, musculoskeletal, vascular, central nervous system, and gastrointestinal manifestations. Behçet’s disease is more prevalent in some regions and populations mainly present along the ancient Silk Road, from the Mediterranean Sea to East Asian countries, including France, Tunisia, Turkey, Israel, Iran, Korea, and Japan [1]. BD has a higher prevalence in these countries (2–420 per 100,000) when compared to the USA and Europe (0.1–7.5 per 100,000). Similarly, a more severe disease spectrum, such as ocular, vascular, and central nervous system inflammation is commonly observed in these regions. Ethnicity and gender are accepted as the major factors affecting the prevalence and manifestations of the disease [2]. However, environmental factors, such as infectious agents ( *Streptococcus* spp., Herpes simplex virus), food, hormones, etc., have also been implicated in the etiopathogenesis of BD, driving pathogenic innate and adaptive immune dysregulation [3–5]. However, relative contributions of genetic or environmental factors to the disease pathogenesis are still unclear. 

About 10 years ago, it was first suggested that the incidence and severity of BD might be declining worldwide [6]. From this perspective, the recent data on the epidemiology of BD and some the environmental factors associated with epidemiological changes will be discussed herein.

## 2. Recent epidemiological studies of BD

Recent studies have supported the wide difference in BD incidence among different populations [7]. One method to investigate the role of genetic vs. environmental factors in BD pathogenesis is to assess the prevalence of BD in a genetically homogeneous population living in two different countries. Studies on Turkish populations living in Turkey vs. European countries are the most comparable for this purpose [1,7], as Turkish populations rarely have interracial marriages in Europe (approximately 5%) [8]. The range of BD prevalence in mainland Turkey (Anatolia) is in the range 70–420/100,000 [7]. As in a previous study from Berlin [9], Kappen et al. recently demonstrated a low prevalence of BD in the Amsterdam region (71/100,000) among the Turkish population [10]. However, both of these studies were census surveys, which depended on hospital/medical data records, whereas the studies performed in Turkey were sample surveys questioning/examining patients directly. A subgroup analysis of 45 reports published between 1974 and 2015 demonstrated a significant difference for prevalence between a sample survey design (82.5/100,000) when compared to a census design (3.6/100,000). According to a metaregression analysis, a study design is identified as an independent covariate significantly affecting the prevalence of BD. Reflecting this methodological issue, in sample surveys, up to 95% of the cases are newly diagnosed, mucocutaneous-limited cases, suggesting that sample surveys perform in the general population to detect milder cases, which are possibly not present in hospital-based census surveys [7]. 

With these methodological issues, it was previously claimed that the only clear clue to whether environmental factors predispose to or modify the disease course can be shown in the longitudinal follow-up of BD patients in an ethnically homogenous, nonimmigrant population with a stable health reimbursement system covering most of the country population [6]. This type of data recently emerged from East Asian countries, such as Korea and Japan. In a recent study using the Korean National Health Insurance Claims Database, covering over 50 million of the Korean population, Lee et al. observed that the incidence of BD had decreased from 7.47/100,000 in 2006 to 2.51/100,000 in 2015 [11]. 

Although epidemiological (community-based data not available), recent data from Japan also suggested that the frequency of BD was decreasing among hospital-based surveys of uveitis patients (23.2% in 1981–1983 vs. 6.2% in 2002), with the prevalence dropping to 7.5/100,000 in 1990 from 8.9/100,000 in 1984 [12–14]. The number of new patients on Hokkaido Island (served with only 1 referral uveitis center) was reported as 83 between 1994 and 2003 when compared to 152 between 1984 and 1993 [15]. The decreasing trend for BD among uveitis patients in Japan was reported to have continued in a recent series [16]. In a series from Taiwan, again with a good reimbursement system, the prevalence of BD among nonanterior uveitis patients decreased from 13.3% in 1991–2000 to 9.5% in 2001–2014 [17]. On the other hand, BD is still the main cause of uveitis (24.9%) among patients followed-up in tertiary uveitis centers in Turkey [18]; however, there might be a decreasing trend compared to a decade ago (32.1%) [19]. 

Another aspect is a decline in the severity of BD in Japan, as the disease is becoming milder with less frequent ocular attacks and vision loss [14]. A lower risk of losing vision was also reported in male patients from Turkey who were diagnosed in the 1990s when compared to patients from the 1980s [20]. Very similar data were also reported from the National Eye Institute [21]. Another trend observed in Japan was the decreasing frequency of complete-type of BD, associated with less vascular, central nervous system, and ocular disease in patients presented after 2008 when compared to pre-2000 [22]. Similar observations of less-severe uveitis and complete-type BD was also reported from Korea in a series followed in the 1990s when compared to the 2000s [23,24]. As expected, with better health coverage, BD patients have been diagnosed earlier and treated better in recent years, and researchers usually explain this trend with earlier and more aggressive treatment approaches. However, a similar trend observed in East Asian countries with stable, good health coverage suggested that the epidemiology might also be changing to a less prevalent and severe disease spectrum for BD. 

## 3. Environmental factors associated with the change in BD epidemiology

The change of BD incidence in populations such as Japan and Korea, which are accepted as fairly stable in genetic/social factors, can be explained mainly by environmental factors. In this context, the association of BD with infections and allergy needs close scrutiny, as they might be the two major environmental factors that have changed in recent years [5].

Oral health, as a part of general health, is affected by barriers to oral health services and personal risk factors [25], as well as the oral ulcer pattern in BD [26] (Figure). Therefore, country-based differences might be seen. Recent studies from the current group and others have demonstrated that BD patients have poor-oral health, demonstrated by impaired dental and periodontal indices, such as caries, loss of teeth, and gingival/periodontal scores [5,26–28]. However, the main issue in these studies was the difficulty in determining the role of persistent oral ulcer presence on the maintenance of oral hygiene, namely brushing and dental floss practices. Despite these difficulties, with better statistical approaches, Mumcu et al. recently demonstrated that both dental caries and the need for tooth extraction, as focal infection focus, were found to be mediators of disease severity. In addition, male gender as a well-known severity factor was also reapproved in their analysis [29]. Oral interventions were also shown to decrease oral ulcer presence in BD patients in a longitudinal 6-month, prospective study [30]. Antibiotics, such as penicillin [31] and azithromycin [32], have been shown to decrease mucocutaneous symptoms, especially folliculitis lesions, and decrease the healing time of oral ulcers and scores of plaque indices [32].

**Figure:**
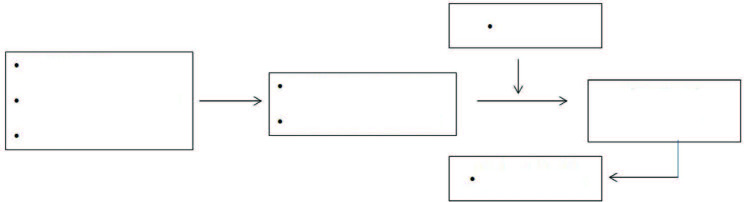
Barriers to oral health services and risk factors for oral health in BD.

Among other environmental factors, smoking, stress, various foods associated with histamine release, and allergies have been implicated as the triggering or suppressing factors for BD incidence and severity [33–35], which were discussed in more detail by Mumcu et al. [5]. However, none of these factors can explain the whole spectrum of the changing epidemiology of BD.

In conclusion, a decline in BD incidence and change in the disease spectrum to less severe manifestations was reported first from Japan and Korea, but requires confirmation in other populations. If this is a worldwide trend, a better understanding of the environmental etiological factors, especially infections associated with the disease onset or relapses, may lead the way to new therapeutic approaches for BD, which is still a disease with significant morbidity, mortality, and work limitations [36].
